# *In vitro *assessment of alendronate toxic and apoptotic effects on human dental pulp stem cells

**DOI:** 10.22038/IJBMS.2018.22877.5816

**Published:** 2018-09

**Authors:** Solmaz Pourgonabadi, Ahmad Ghorbani, Zahra Tayarani Najarn, Seyed Hadi Mousavi

**Affiliations:** 1 Department of Oral and Maxillofacial Surgery, Dental Research Center, School of Dentistry, Mashhad University of Medical Sciences, Mashhad, Iran; 2 Pharmacological Research Center of Medicinal Plants, Mashhad University of Medical Sciences, Mashhad, Iran; 3 Medical Toxicology Research Center, Mashhad University of Medical Sciences, Mashhad, Iran; 4 Department of Pharmacodynamics and Toxicology, School of Pharmacy, Mashhad University of Medical Sciences, Mashhad, Iran

**Keywords:** Alendronate, Apoptosis, Bisphosphonates, Human dental pulp stem cells, Proliferation

## Abstract

**Objective(s):**

Osteonecrosis of the jaw, as an exposed necrotic bone in the oral cavity, is one of the adverse effects of bisphosphonates, which have an affinity for bone minerals. This study investigates the cytotoxic effects of alendronate (ALN) as a nitrogen-containing bisphosphonate, on human dental pulp stem cells (hDPSCs).

**Materials and Methods:**

The mesenchymal stem cells (MSCs), obtained from third molar tooth pulps were characterized by immunophenotyping assay in order to identify surface markers to evaluate their expression. To detect multipotency hDPSCs, they were differentiate into osteocytes and adipocytes. Cell proliferation was measured by MTT assay. PI staining of DNA fragmentation by flowcytometry (sub-G1 peak) was performed for determination of apoptotic cells and Bax, Bcl-2, and cleaved caspase 3 expressions. Protein expression was detected by Western blotting.

**Results:**

As the results revealed, ALN decreased viable cells (in 0.8–100 µM) after 72 hr and 168 hr (*P*<0.001), significantly. ALN could lower cell proliferation in hDPSCs in a concentration and time-dependent manner. Sub-G1 peak as an indicator of flowcytometry histogram of treated cells by ALN, showed apoptosis was involved in ALN-induced cytotoxicity. Expressions of cleaved caspase 3 and Bax protein, as pro-apoptotic proteins, were increased and Bcl-2 protein as anti-apoptotic protein was decreased in response to increases in the concentration of ALN (0.8–25 µM).

**Conclusion:**

Long-term effects of ALN on cell proliferation and apoptosis in hDPSCs can result in either initiation or potentiation of ALN-induced osteonecrosis.

## Introduction

Bisphosphonates are a family of synthetic compounds of naturally occurring pyrophosphates, which are used to treat and prevent deformed bone metabolism diseases including osteoporosis, Paget’s, and tumor-induced bone disease (1-2). Strong affinity of bisphosphonates to the hydroxyapatite crystals makes them stop osteoclast-mediated bone disease either through inhibition of osteoclast activation or function. ALN is the first amino bisphosphate and a third generation drug marketed as Fosamax (Merck &Co, Inc., Whitehouse Station, NJ, USA). Presence of nitrogen in the structure of ALN makes it stronger than the other oral bisphosphonates (2-5). 

There is also evidence that ALN has an effect on proliferation of MG63 osteoblast-like cells in concentrations ≤10^-5^ M. In contrast, ALN does not have any toxic effect on osteoclasts in rabbits either in vivo or in vitro. Also, its use in cancer-associated hypercalcemia does not have any serious side effect. However, it has the potential to prevent canine osteosarcoma tumor growth (5, 6). Osteonecrosis of the jaw is one of rare side effects of bisphosphonates that are used clinically for inhibition of skeletal-related obstacles in malignant bone diseases (7). Cytotoxic effect of ALN on oral keratinocytes is thought to be involved in the initiation osteonecrosis of the jaw. The inhibitory effect of ALN on stem cells and other cell types of the oral cavity may be involved in the developing osteonecrosis in patients receiving ALN (8, 9). ALN reduces viability and proliferation of human epithelial cells and gingival and periodontal ligament fibroblasts (10). In the development of postnatal tissue, hDPSCs play an effective part as a promising cell source for tooth and bone-tissue engineering (11-14). 

Although bisphosphonates disturb odontogenesis and make defects in dental structures, few studies have reported about bisphosphonates effect on tooth defects that are accessible.

Thus, this study was designed to evaluate the dose-response effect of ALN on the proliferation of hDPSCs. Meanwhile, the role of apoptosis was also explored in ALN-induced toxicity. 

## Materials and Methods

***Chemicals and reagents***

Alizarin Red, ascorbic acid, dexamethasone, β-glycerophosphate, penicillin-streptomycin solution, a collagenase from Clostridium histolyticum, 3-isobutyl-1-methylxanthine (IBMX), 3-(4,5-Dimethyl-2-thiazolyl)-2,5-diphenyl-2H-tetrazolium bromide (MTT), propidium iodide, and bicinchoninic acid protein assay kit were purchased from Sigma-Aldrich (St. Louis, MO, USA). Fetal bovine serum (FBS), Dulbecco’s Modified Eagles Medium (DMEM), and trypsin were obtained from Gibco (Grand Island, NY, USA). Dimethyl sulfoxide and Oil Red O were purchased from Merck (Darmstadt, Germany). Fluorescein isothiocyanate-conjugated antibodies against CD29, CD34, CD44, and CD45 were obtained from AbD Serotec (Raleigh, USA). Phycoerythrin-conjugated antibodies against CD90 and CD105 were purchased from Novus Biological (Littleton, CO, USA) and Exbio (Czech Republic), respectively. ALN from Merck Pharmaceuticals Corporation was kindly provided by Arastoo Com. (Iran). Antibodies against β-actin, cleaved caspase 3, Bcl-2, Bax, and horseradish peroxidase-conjugated goat anti-rabbit IgG were obtained from Cell Signaling Technology (Danvers, USA) (15).

***Isolation of hDPSCs***

The hDPSCs were isolated from teeth of healthy subjects who were undergoing oral surgery (wisdom tooth extraction) in the Clinic of Dentistry, Mashhad University of Medical Sciences (Iran). The Ethics Committee of Mashhad University of Medical Sciences approved procedures of this study. To expose the pulp chamber, they were cleaned and cut. The pulp tissue were removed from the chamber by special dental instruments and cut into small pieces (≈ 2–3 mm) then stem cells were obtained by the explant culture method (16). Briefly, the tissue pieces were explanted into a culture flask and their surfaces were covered with FBS. The explants were incubated overnight at 37 ^°^C in an atmosphere of 5% CO_2_. Then, FBS was changed by high glucose DMEM supplemented with 20% FBS, penicillin (100 units/ml), and streptomycin (100 µg/ml), and the cultures were observed until the fibroblast-like cells appeared and expanded around the tissue pieces. In the subconfluent state, the cells were harvested and expanded further through 3 passages.

***Detection of CD markers in hDPSCs***

*Flow cytometric analysis assessed surface markers that are expressed on stem cells*

The isolated hDPSCs at passage 4 were detached from the culture flask by trypsin-EDTA, centrifuged at 2000 rpm for 5 min, and resuspended in phosphate buffer solution containing 2% FBS. Then, the cells were incubated with antibodies against CD29, CD34, CD44, CD45, CD90, and CD105 for 30 min at 4 °C. After washing with phosphate buffer, the cells were suspended in 500 µl of the buffer containing 2% FBS and analysis was performed using FACSCalibur flow cytometer (BD Biosciences) (15).

***Characterization of the multipotency of hDPSCs***

Characterization of the multipotency of hDPSCs mweans evaluating their ability to differentiate into adipocyte and osteoblast lineages. Briefly, hDPSCs were seeded in 6-well plates containing DMEM supplemented with 10% FBS and penicillin/streptomycin and cultured to reach 80% confluency for differentiation testing. Then the culture medium was replaced with adipogenic or osteogenic differentiation media. Adipogenic medium comprised DMEM supplemented with 3% FBS, 1 μM dexamethasone, and 0.2 μM insulin (16). The adipogenic medium was exchanged every 3 days and the cells were maintained in this medium for 3 weeks. Intracellular triglyceride droplets were stained by Oil Red O for adipogenesis.

 The cells were fixed with 10% formalin and then incubated for 20 min with Oil Red O solution (17). Then, the cells were washed three times with distilled water and photographed using an inverted microscope.

Osteoblastic differentiation medium consisted of DMEM supplemented with 10% FBS, 10 μg/ml ascorbic acid, 5 mM β-glycerol phosphate, and 0.1 μM dexamethasone (18). This medium was exchanged every 3 days and the cells were maintained for 4 weeks. Osteogenic differentiation was confirmed by Alizarin Red and alkaline phosphatase staining, which stain extracellular calcium deposits and the alkaline phosphatase enzyme, respectively. The cells were fixed with 10% formalin and then incubated for 5 min with 2% Alizarin Red solution, or alkaline phosphatase reagent. Then, the cells were washed three times with distilled water and photographed using an inverted microscope.

**Figure 1:**
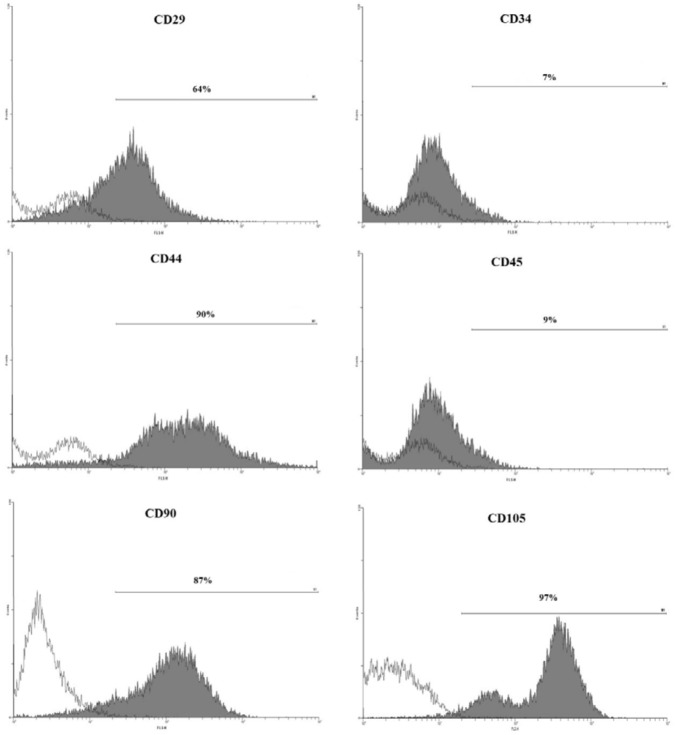
Flowcytometric analysis of cell-surface markers in human dental pulp stem cells (hDPSCs) derived from human third molar teeth. This analysis revealed that human dental pulp stem cells (hDPSCs) at passage 4 expressed MSC markers CD29, CD44, CD90 and CD105, and were negative for hematologic markers CD34 and CD45

**Figure 2:**
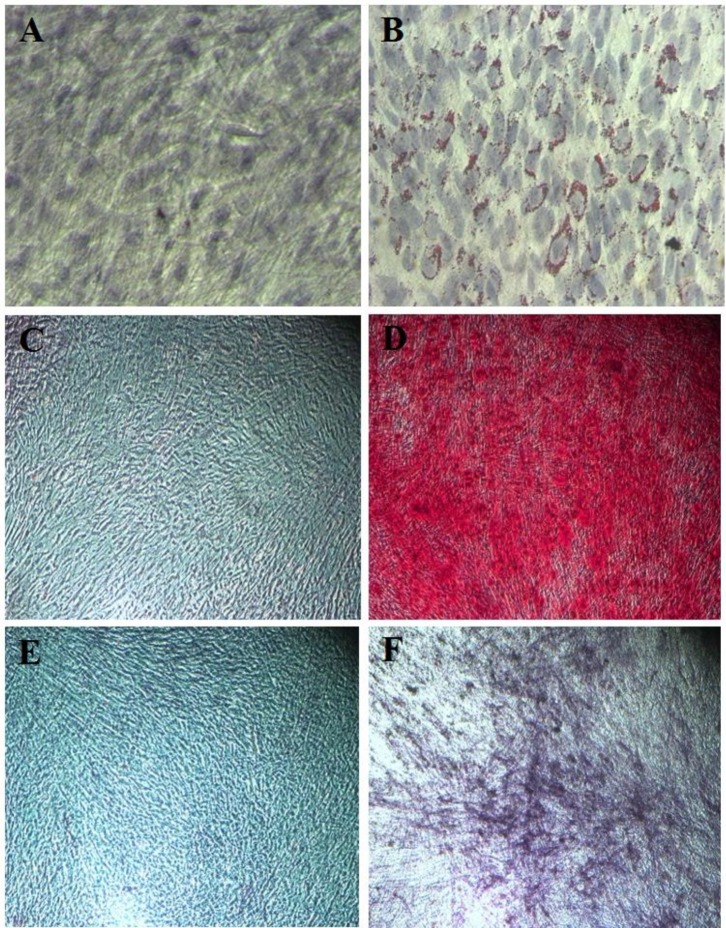
Differentiation of human dental pulp stem cells (hDPSCs) to adipocyte and osteocyte lineages. a: Oil Red O staining of human dental pulp stem cells (hDPSCs) cultured 3 weeks in control standard medium (magnification ×200); b: Oil Red O staining of human dental pulp stem cells (hDPSCs) cultured 3 weeks in adipogenic differentiation medium (magnification ×200); c: Alizarin Red staining of human dental pulp stem cells (hDPSCs) cultured 4 weeks in control standard medium (magnification ×100); d: Alizarin Red staining of human dental pulp stem cells (hDPSCs) cultured 4 weeks in osteogenic differentiation medium (magnification ×100); e: alkaline phosphatase staining of human dental pulp stem cells (hDPSCs) cultured 4 weeks in control standard medium (magnification ×100); f: alkaline phosphatase staining of human dental pulp stem cells (hDPSCs) cultured 4 weeks in osteogenic differentiation medium (magnification ×100)

**Figure 3:**
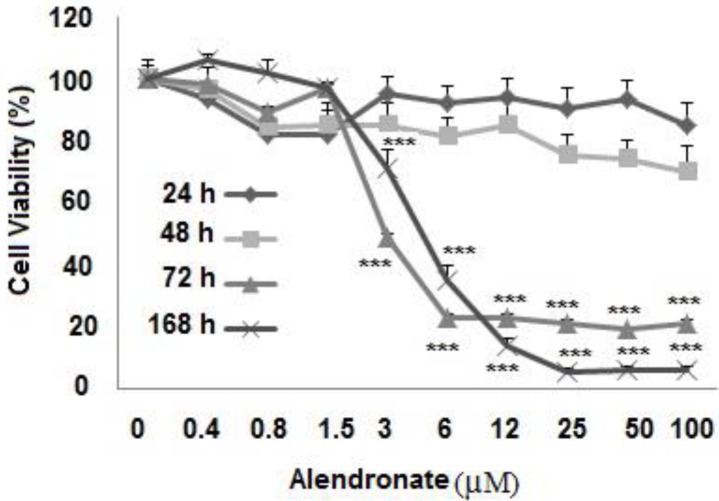
Effect of alendronate (ALN) on proliferation of human dental pulp stem cells (hDPSCs), the percent of viable cells was measured versus control cells (concentration of 0). Data are mean±SEM (n=9). *P<0.05 versus concentration of 0; ***P<0.001 versus control

**Figure 4:**
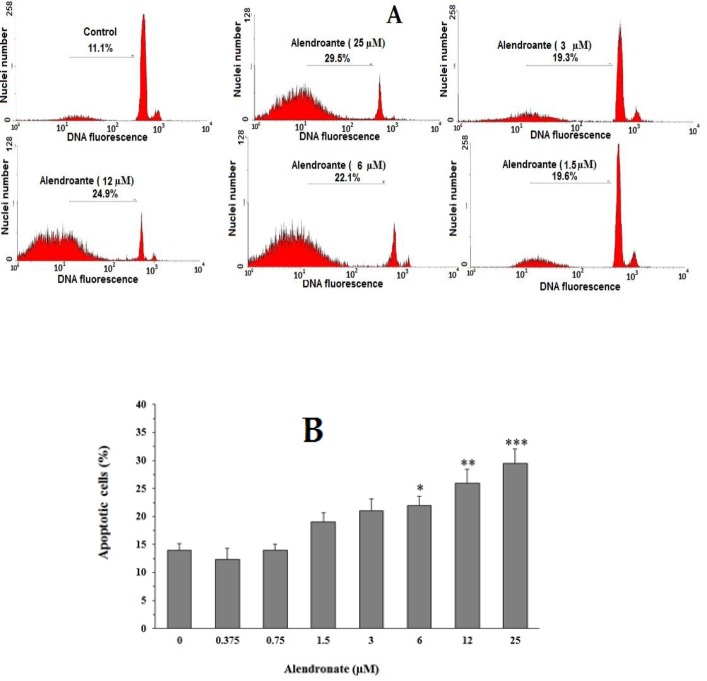
Effect of alendronate (ALN) on apoptosis of human dental pulp stem cells (hDPSCs). The cells were treated for 72 hr with different concentrations of alendronate (ALN) and then stained with propidium iodide, apoptotic cells have been shown by the sub-G1 region which shows DNA fragmentation. a: representative histogram of the fluorescence intensity of propidium iodide-stained human dental pulp stem cells (hDPSCs); b: quantitative analysis of human dental pulp stem cells (hDPSCs) apoptosis. *P<0.05, **P<0.01, ***P< 0.001 versus control cells

**Figure 5:**
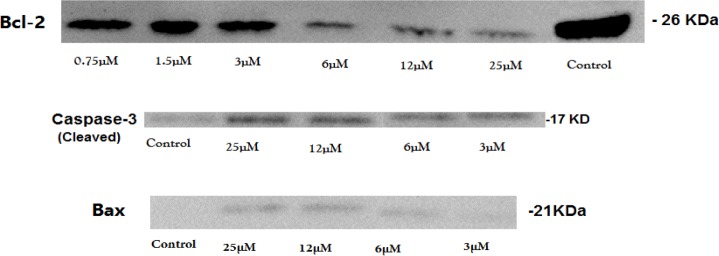
Effect of alendronate (ALN) on the levels of pro-apoptotic proteins (Bax and caspase-3) and anti-apoptotic protein (Bcl-2) in human dental pulp stem cells (hDPSCs), Western blotting analyses for Bax, cleaved caspase-3, and Bcl-2 proteins were performed on human dental pulp stem cells (hDPSCs) treated for 72 hr with alendronate (ALN)

***Cell proliferation assay***

The hDPSCs were seeded in 96-well plates overnight and then cultured for 24 hr, 48 hr, 72 hr, and 7 days in DMEM containing 10% FBS and different concentrations of ALN (0.2–100 µM). Then, MTT reagent was added to each well (at the final concentration of 0.05%) and the cells were maintained in an atmosphere of 5% CO_2_ at 37 ^°^C. After 3 hr , the supernatant was removed and the formazan crystals were dissolved in 100 µl dimethyl sulfoxide. The optical density of formazan dye was read at 540 and 630 nm using a StatFAX3200 plate reader (19, 20). The MTT assay was performed three times in triplicate.

***Apoptosis assay***

The hDPSCs were seeded in 12-well plates overnight and then cultured for 72 hr in DMEM containing 10% FBS and different concentrations of ALN (0-25 µM). After permeabilization and staining these cells with 500 µl propidium iodide reagent (5 mg propidium iodide, 100 mg sodium citrate, and 100 µl Triton-X 100 in 100 ml distilled water), which was added to each well, for 30 min the plates were maintained at 37 ºC (21, 22). Apoptotic cells were detected using propidium iodide nuclear fluorescence intensity of the cells by flow cytometry.

***Western blotting analysis***

 hDPSCs treated with different concentrations of ALN (3–25 µM) for 72 hr were harvested and suspended in a lysis buffer containing 50 mM Tris-HCl (pH 7.4), 150 mM NaCl, 1% Triton-X 100, 1 mM EDTA, 0.2% SDS, 1% protease inhibitor cocktail, and 1 mM phenylmethylsulfonyl fluoride for 45 min on ice.

The protein concentrations for each sample were determined using BCA assay kit and the cell lysate was centrifuged at 10000 rpm for 20 min at 4 ^°^C. Equal volumes of proteins from each sample were loaded to 12.5% SDS-PAGE (w/v) and electrophoresed. The proteins were transferred to a polyvinylidene fluoride membrane and subjected to immunoblotting using primary antibodies against β-actin, cleaved caspase-3, Bcl-2, and Bax. A horseradish peroxidase-conjugated goat anti-rabbit IgG secondary antibody made the bounds visible and chemiluminescence system detected them (23). 

***Statistical analysis***

One-way analysis of variance and *post hoc* Dunnett’s multiple comparison tests were used to analyze these data in IBM SPSS statistical software (ver. 20). A probability level of *P*<0.05 was statistically significant in mean ± SEM results.

## Results

***Characterization of hDPSCs***

The MSCs Markers CD29, CD44, CD90, and CD105 were positive in hDPSCs while CD34 and CD45, as hematologic makers, were negative, as revealed by flow cytometric analysis (Figure 1). Cells with pluripotent capacity were cultured in adipocyte and osteocyte differentiating media. To confirm the capability of isolated hDPSCs to differentiate into adipocyte lineage, Oil Red O staining showed intracellular lipid droplets (Figures 2A and 2B). Also, osteogenic differentiation ability of hDPSCs has been shown with alkaline phosphatase and Alizarin Red S staining (Figures 2C–2F).

***Effect proliferative alendronate on hDPSCs***

As shown in Figure 3, none of the ALN concentrations decreased proliferation of hDPSCs in 24 hr and 48 hr. When the cells were incubated for 72 hr and 7 days in the presence of ALN, concentrations of up to 3 µM significantly decreased the percent of viable hDPSCs (*P*<0.001). After 72 hr, in the presence of 3, 6, 12, 25, 50, and 100 µM of ALN, viability of hDPSCs decreased from 100 ± 6.2% (control) to 49 ± 7.2%, 23 ± 3.3%, 23 ± 3.6%, 21 ± 3.7%, 19 ± 1.2%, and 21 ± 2.6, respectively (*P*<0.001). In similar concentrations, after 7 days treatment with this drug, viability of hDPSCs significantly decreased from 100±1.6 (control) to 71±6.2%, 35±5%, 14±2.5%, 5.5±1% ,6±1%, and 7±1.2%, respectively (*P*<0.001). 

The dose inducing 50% cell growth inhibition (IC50) against hDPSCs for ALN in 72 hr and 7 days were calculated to be 4 μM.

***Alendronate induces apoptosis on hDPSCs***

Apoptotic effect after 72 hr incubation with various concentration of ALN has been shown in Figure 4. To show the histogram of fluorescence intensity of propidium iodide-stained hDPSCs, DNA fragmentation produced the Sub-G1 region related to apoptotic hDPSCs (Figure 4A). Apoptotic hDPSCs in Sub-G1 phase were increased by adding increased concentrations of ALN to the cell culture medium in a concentration-dependent manner.

 In the presence of 6, 12, and 25 µM of ALN, the percent of apoptotic hDPSCs increased from control (untreated cells) 14 ± 1.2%, 22 ± 1.6%, 26 ± 2.4%, and 29.5 ± 2.6%, respectively (*P*<0.05 – *P*<0.001) (Figure 4B).

***Evaluation of pro-apoptotic and anti-apoptotic proteins***

 The level of pro-apoptotic proteins cleaved caspase-3 and Bax and anti-apoptotic protein Bcl-2 were determined by Western blotting analysis. 

Treatment of hDPSCs with various concentrations of ALN (3–25 µM) for 72 hr increased the level of cleaved caspase-3 and Bax, while significantly decreasing the level of Bcl-2 in comparison with untreated cells (Figure 5).

## Discussion

Alendronate as a nitrogen-containing bisphosphonate has been widely examined for proliferation and viability of various cell types in previous studies (10, 24-26).

 Yet, the likely cytotoxic and apoptotic effects of ALN on hDPSCs has not been examined in other studies. In this study, hDPSCs from healthy subjects were isolated and characterized by determining expression of stem cell surface markers and multipotency for adipocyte and osteocyte differentiation of hDPSCs. To investigate the effect of ALN on hDPSCs, this study was designed. The results indicated when ALN was incubated for 72 hr and 168 hr it could decrease cell proliferation of hDPSCs. ALN has an anti-proliferative character in hDPSCs in a concentration and time-dependent manner.

This may highlight the fact that long-term ALN exposure could induce cytotoxicity in hDPSCs in vitro. In our study, we can assume when ALN is used in a clinical trial, following animal testing up to 48 hr, it induces toxicity in various cells in the oral cavity specially hDPSCs. It seems likely that this cytotoxicity effect has a potential role in the initiation of osteonecrosis of the jaw. In our study, reduced cell proliferation by ALN was accompanied with increased sub-G1 phase as an apoptosis indicator in the normal cell cycle.

Also, Western blotting data confirmed that in the presence of ALN the protein level of Bcl-2 was reduced, but Bax and cleaved caspase-3 were increased, which led to hDPSCs apoptosis. The effects of ALN on proliferation and apoptosis of hDPSCs were started from concentrations as low as 3 and 6 µM, respectively.

Correia *et al.* revealed that ALN at a concentration of 10^-6^ M diminished the viability of human periodontal ligament cells and at a concentration 10^-5^ M changed cell morphology (24). Moreira *et al.* reported that ALN mixed with paste had a toxic effect on endothelial cells at a concentration of 0.6×10^-5^ M (25). 

However, Sato *et al.* estimated that ALN may reach concentrations of 0.5 M in resorption space in bones, there is unclear evidence that similar concentrations can be found around oral mucosal cells for any period of time (26). 

Clinically, these concentrations can be related to patients who receive ALN 70 mg weekly.

 As its plasma concentration is about 0.116 µM following oral administration, its bioavailability is approximately 0.7% (27).

In this study, effects of ALN were significant only when this drug had long-term hDPSCs exposure of more than 72 hr. There are several reports on the effects of ALN in different concentrations on cell viability and functions of various tissues. In agreement with the present observation, in vitro studies revealed that ALN reduced proliferation of gingival fibroblasts (10, 24) and oral keratinocytes (28). 

Indeed, these results agree with former investigations indicating ALN has toxicity in cells of various kinds of tissues of the mouth (24, 25, 29). It is well known that MSCs play important roles in postnatal tissue development, tissue repair, and disease modification (30). Therefore, ALN may have a negative impact on these roles of stem cells. In line, research showed that ALN diminished bone formation within root socket in the jaw (31).

## Conclusion

Taken together, based on the results long-term ALN exposure induces anti-proliferative and pro-apoptotic effects on hDPSCs, which may have a negative impact on dental pulp and be involved in initiation or potentiation of ALN-induced osteonecrosis. So far no exact intracellular mechanisms that are responsible for the anti-proliferative actions of ALN are clear and should be revealed in future studies.
